# Absence of TolC Impairs Biofilm Formation in *Actinobacillus pleuropneumoniae* by Reducing Initial Attachment

**DOI:** 10.1371/journal.pone.0163364

**Published:** 2016-09-28

**Authors:** Ying Li, Sanjie Cao, Luhua Zhang, Jianlin Yuan, Gee W. Lau, Yiping Wen, Rui Wu, Qin Zhao, Xiaobo Huang, Qigui Yan, Yong Huang, Xintian Wen

**Affiliations:** 1 Research Center of Swine Diseases, College of Veterinary Medicine, Sichuan Agricultural University, Chengdu, 611130, China; 2 Department of Pathobiology, University of Illinois at Urbana-Champaign, Urbana, Illinois, United States of America; National Institute of Technology Rourkela, INDIA

## Abstract

*Actinobacillus pleuropneumoniae* is the etiologic agent of porcine contagious pleuropneumonia, a major cause of economic loss in swine industry worldwide. TolC, the key component of multidrug efflux pumps and type I secretion systems, has been well-studied as an exit duct for numerous substances in many Gram-negative bacteria. By contrast, little is known on the role of TolC in biofilm formation. In this study, a Δ*tolC* mutant was used to examine the importance of TolC in biofilm formation of *A*. *pleuropneumoniae*. Surface attachment assays demonstrated the essential role of TolC in initial attachment of biofilm cells. The loss of TolC function altered surface hydrophobicity, and resulted in greatly reduced autoaggregation in Δ*tolC*. Using both enzymatic treatments and confocal microscopy, biofilm composition and architecture were characterized. When compared against the wild-type strain, the poly-β-1, 6-N-acetyl-D-glucosamine (PGA), an important biofilm matrix component of *A*. *pleuropneumoniae*, was significantly reduced at the initial attachment stage in Δ*tolC*. These results were confirmed by mRNA level using quantitative RT-PCR. Additionally, defective secretion systems in Δ*tolC* may also contribute to the deficiency in biofilm formation. Taken together, the current study demonstrated the importance of TolC in the initial biofilm formation stage in *A*. *pleuropneumoniae*. These findings could have important clinical implications in developing new treatments against biofilm-related infections by *A*. *pleuropneumoniae*.

## 1. Introduction

*Actinobacillus pleuropneumoniae* is a Gram-negative bacterium in the family of *Pasteurellaceae*. It is the etiologic agent of porcine contagious pleuropneumonia, a significant respiratory disease in swine, with serious economic losses to the swine industry worldwide [1]. To date, there have been a lot of studies on virulence factors of *A*. *pleuropneumoniae* that contribute to colonization and lung infection. Among them, biofilm formation mediates in vivo colonization of host tissues [2]. However, not much is known about the mechanism of biofilm formation in *A*. *pleuropneumoniae*. A better understanding of the mechanisms that influence or regulate the biofilm formation would potentially improve antibiofilm strategies in this pathogen.

Biofilms are multicellular communities that cells are encased in a self-produced extracellular matrix, which could attach to biotic or abiotic surfaces. Biofilms protect pathogenic bacteria from deleterious agents and other stresses within their environment, rendering them difficult to treat. Therefore, it is not surprising that biofilms of pathogens are important for chronic infections in human and animals [3]. The biofilm matrix is known as extracellular polymeric substances (EPS), of which exopolysaccharides, extracellular DNA (eDNA) and extracellular proteins are the most important constituents. Exopolysaccharides vary greatly in their compositions, and are identified as the framework of biofilm architecture in many bacteria and play significant roles in intercellular adhesion, biofilm maturation and dispersal of biofilm cells [4]. eDNA is also a structural component of biofilm matrix in some bacterial species and often plays a crucial role in establishment and maintenance of bacterial biofilms [5]. Various kinds of proteins can be found in the biofilm matrix, including extracellular enzymes, structural proteins and proteinaceous appendages [6]. These extracellular proteins are often involved in the integrity and stabilization of the polysaccharide matrix network [7]. Collectively, the EPS provide the scaffold of the three-dimensional biofilm architecture and play crucial roles in surface adhesion and cohesion in the biofilm [7].

The development of biofilms varies between bacterial species and different living conditions. In general, biofilm formation is initiated by the attachment of cells to a surface and production of EPS, followed by recruitment of additional bacteria, and proliferation to form microcolonies which eventually develop into a mature biofilm [8]. During the early stages, a variety of extracellular adhesive substances such as eDNA, adhesins, as well as bacterial surface structures such as flagella, pili and curli fimbriae are employed to initiate the attachment. Bacterial cell surface hydrophobicity could affect the aggregation of microcolonies, and the excretion of extracellular matrix further strengthens the adherence. As the microcolony grows, bacteria switch to a distinct pattern of gene expression and are surrounded by EPS and intercellular signaling molecules, resulting in the maturation of biofilms with complex three dimensional structures [3,5].

In *A*. *pleuropneumoniae*, biofilm formation is prevalent among field isolates [9], with the extracellular polymer of PGA as a key component of the biofilm matrix [10]. Dispersin B protein, encoded by *dspB*, is a PGA-hydrolyzing enzyme that could dissolve the biofilm matrix and disperse cells. Multiple genes are involved in the biofilm formation or regulation, including the sigma factor σ^E^ [11], serine protease AasP [12], RNA chaperone Hfq [13], ClpP protease [14] and the recently identified O-antigen of LPS [15]. Moreover, transcriptome analysis of biofilm cells has identified more proteins as the potential regulator or cues that affect biofilm formation in *A*. *pleuropneumoniae* [16]. Despite these advancements, knowledge regarding the regulation of biofilm development processes remains limited.

TolC is an outer membrane channel component of multidrug efflux pumps and type I secretion systems in *Escherichia coli*. Numerous studies have revealed the importance of TolC in drug resistance and bacterial virulence [17]. Recently, the correlation between efflux pump genes, including TolC, and biofilm formation has attracted an increasing interest. Studies have showed that efflux pumps play key roles in the biofilm growth of *E*. *coli*, *Klebsiella pneumoniae*, *Staphylococcus aureus*, and *Salmonella typhimurium*, and efflux pump inhibitors have been identified as efficient antibiotic synergists against biofilm-related infections of these pathogens [18,19]. In our recent study, a protein TolC1 was confirmed, now named TolC, to be required for biofilm formation of *A*. *pleuropneumoniae* without a substantial growth inhibition (Unpublished data). However, the mechanism by which TolC regulates biofilm development remained poorly understood.

The objective of this study was to determine the link between TolC and biofilm development of *A*. *pleuropneumoniae* using a Δ*tolC* mutant. The inactivation of TolC was found to be deficient in initial surface attachment step during biofilm formation. Subsequent assays pinpointing the crucial role of TolC in initial attachment was carried out by analyzing the bacterial surface hydrophobicity, biofilm composition, and PGA production.

## 2. Results

### 2.1 Inactivation of TolC impairs biofilm formation in *A*. *pleuropneumoniae*

**Fig 1:**
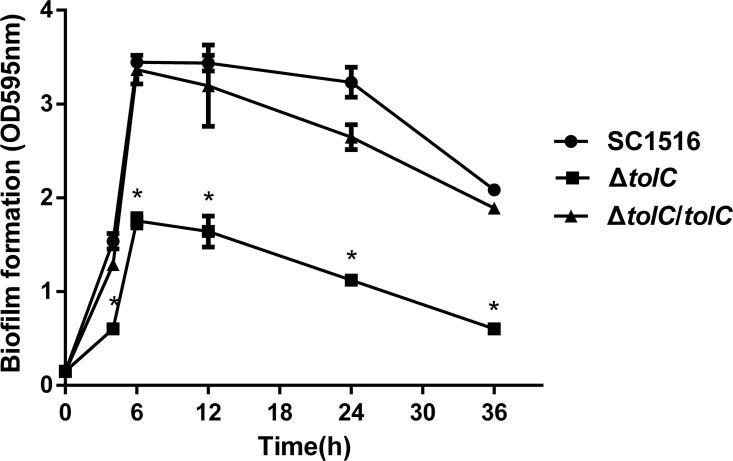
Effects of *tolC* deletion on biofilm formation of *A*. *pleuropneumoniae*. Biofilm formation was determined at various time points (0, 4, 6, 12, 24 and 36h) by crystal violet staining using static broth cultures of WT SC1516, Δ*tolC* and the genetically-complemented strain Δ*tolC*/*tolC*. Data are means ± standard deviations (SDs) of three independent experiments with three replicates. Asterisks indicate statistical significance (P< 0.05).

The time course of biofilm biomass for *A*. *pleuropneumoniae* SC1516, Δ*tolC* and the genetically-complemented strain Δ*tolC*/*tolC* were measured to investigate the kinetics of biofilms formation and to determine an appropriate time point to perform further studies. Biofilm biomass was quantified using a crystal violet staining at 0, 4, 6, 12, 24 and 36 h after incubation. As shown in Fig 1, the amount of biofilm in Δ*tolC* mutant was significantly reduced compared to that in the wild-type (WT) strain during the course of experiments. The ability of biofilm formation was restored back to the WT level in Δ*tolC*/*tolC*. The biofilm biomass peaked at 6 and 12 h, and decreased gradually over time in all groups. Based on the time course data, we tentatively designated 6 h as the time point of biofilm being fully formed and 12 h as the biofilm maturation in all subsequent studies.

### 2.2 Inactivation of TolC reduces initial attachment of biofilms in *A*. *pleuropneumoniae*

**Fig 2:**
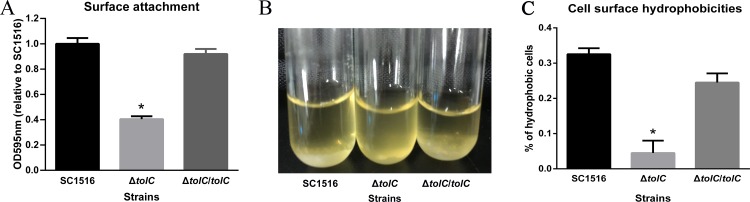
Effects of TolC on initial surface adherence, autoaggregation and cell surface hydrophobicity. (A) The surface attachment assay was determined in 96-well microtiter plates by staining with crystal violet. Attached cells in the well were quantitated by measuring the optical density at 595 nm. Data represents mean values for triplicate wells from three independent experiments. Error bars indicate SDs and the asterisks show significant differences (p< 0.05). (B) The Δ*tolC* cells remained mostly in suspension (middle), while the WT (left) and complemented strain (right) autoaggregated and settled to the bottom of the tubes after 6 h static incubation at room temperature. (C) The surface hydrophobicity of Δ*tolC* cells was significantly reduced when compared against WT. Values are expressed as means ± SE, * *p* < 0.05.

To evaluate whether TolC was involved in the initial surface attachment of *A*. *pleuropneumoniae*, the ability of adherence to the plastic substrate was determined in a 96-well microtiter plate by crystal violet staining. When compared against WT and complemented strains, the Δ*tolC* showed reduced number of attached cells in the well (Fig 2A), which was proportional to the optical density (OD) at 595nm [10]. This result indicated that Δ*tolC* is defective in initial surface attachment step during biofilm formation.

### 2.3 Inactivation of TolC decreases bacterial cell autoaggregation of *A*. *pleuropneumoniae*

The aggregative ability of each strain was determined by the sedimentation rate of the bacterial cells. As shown in Fig 2B, WT and Δ*tolC*/*tolC* cells sedimented to the bottom of the culture tubes, while Δ*tolC* cells remained in suspension. These results showed that Δ*tolC* cells were less adhesive and therefore, showed a reduced autoaggregation phenotype. This finding suggested that the loss of TolC may alter cell surface hydrophobicity of *A*. *pleuropneumoniae*.

### 2.4 Inactivation of TolC decreases cell surface hydrophobicity in *A*. *pleuropneumoniae*

To determine whether inactivation of TolC led to alteration in the cell surface hydrophobicity, the hydrophobicity values were estimated based on the ability to bind with hexadecane. As a result, when compared against WT, a decreased hydrophobicity was observed in the *tolC* mutant, while the Δ*tolC*/*tolC* restored the surface hydrophobicity (Fig 2C). These results suggested that inactivation of TolC decreases cell surface hydrophobicity. The reduced cell hydrophobicity of Δ*tolC* may be one of the explanations for its defectiveness in initial surface attachment.

### 2.5 Inactivation of TolC changes the biofilm composition of *A*. *pleuropneumoniae*

**Fig 3:**
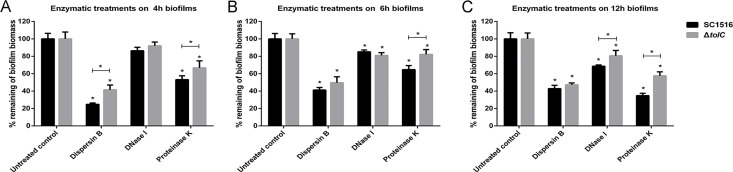
Detachment of biofilm colonies of WT *A*. *pleuropneumoniae* and Δ*tolC* grown in 96-well microtiter plates. Biofilms were cultured for 4h (A), 6h (B) and 12h (C) and treated with dispersin B, DNase I and proteinase K, respectively. The remaining biofilms were quantitated by crystal violet staining. Data represent mean values ± SDs of three independent experiments with three replicates. Results with significant differences when compared to the untreated controls or between two strains were marked with asterisks (P< 0.05).

To determine the components of biofilm matrix of *A*. *pleuropneumoniae* SC1516 and Δ*tolC*, enzymatic treatments were performed using dispersin B, DNase I and proteinase K on biofilms cultured at 4, 6 and 12 h, respectively. As shown in Fig 3, the addition of dispersin B, a glycoside hydrolase that specifically degrades PGA, resulted in significant dispersal of cells from both the WT and the Δ*tolC* mutant biofilms. Digestions with proteinase K also significantly reduced the biofilm formation in all groups, and DNase I showed a significant dispersion effect on biofilms incubated for 6 h and 12 h. These results indicated that PGA was indeed a major component of *A*. *pleuropneumoniae* biofilm matrix, while proteins and eDNA were also involved in the formation of biofilm architecture. The data in Fig 3A showed that, at the initial attachment stage, the Δ*tolC* biofilms were significantly less sensitivity to dispersin B than that of WT strain. This result suggested that the loss of TolC reduced PGA production in early-stage biofilms. Similarly, at all time points analyzed, the biofilms of Δ*tolC* were more resistant to the digestion of proteinase K than WT, suggesting that less extracellular proteins were involved in biofilm matrix in Δ*tolC*.

### 2.6 The loss of TolC changes the biofilm morphology of *A*. *pleuropneumoniae*

**Fig 4:**
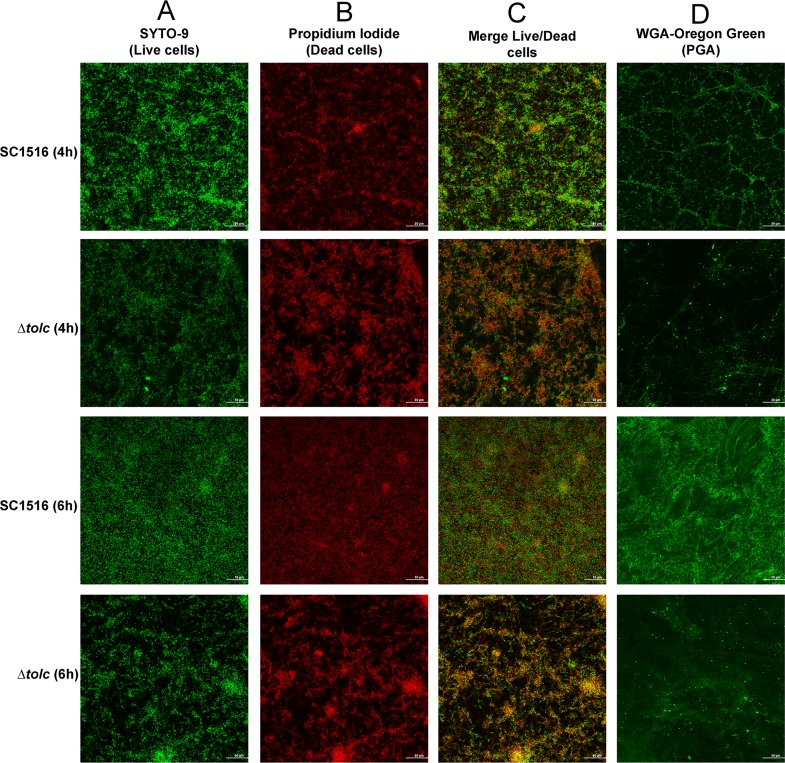
CLSM images of biofilms formed by WT and Δ*tolC*. Both 4-h and 6-h static biofilms cultured in 6-well microtiter plates were stained with the SYTO-9 and propidium iodide (A-C) to label live versus dead cells, or with WGA-conjugated to Oregon green to label PGA (D).

Next, we examined if reduced PGA and extracellular proteins in Δ*tolC* altered its biofilm structures. The biofilms of WT and Δ*tolC* mutant were compared by confocal laser scanning microscopy (CLSM). Biofilms at 4 h and 6 h in microtiter plates were washed and stained with SYTO-9 (Fig 4A) and propidium iodide (Fig 4B) to label the live and dead cells, respectively. Fig 4A showed significant reduction in attached cells of Δ*tolC* at both of these two time points, as indicated by decreased fluorescence intensity of merge images (Fig 4C). The results were consistent with the surface attachment assays (Fig 2A). Besides, a significantly higher proportion of dead cells were observed in Δ*tolC* when compared against WT strain (Fig 4B). These observations suggested that TolC was required to maintain the viability of *A*. *pleuropneumoniae* within a biofilm. The biofilm architecture was further analyzed by using the WGA fluorescent probe that specifically labeled the PGA, the framework of the biofilm architecture. As shown in Fig 4D, Δ*tolC* formed thinner biofilms as seen by scattered fluorescent spots, while the biofilms produced by WT appeared exuberant that diffused across the entire field of view. These results indicated that the loss of TolC significantly changed the morphology of the biofilm when compared to WT. Overall the abundance of biofilms observed with CLSM was consistent with the quantification by crystal violet staining (Fig 2A).

### 2.7 The loss of TolC reduces the transcription of *pgaA* and *cpxR* genes in *A*. *pleuropneumoniae*

**Fig 5:**
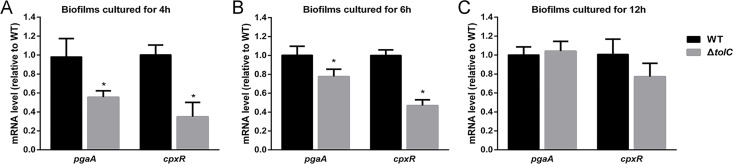
Relative expression of *pgaA* and *cpxR* in biofilms of Δ*tolC* versus WT. The mRNA levels of *pgaA* and *cpxR* genes in biofilms at 4h (A), 6h (B) and 12h (C) were determined by qRT-PCR. Data presented are means of three independent experiments with triplicates. Error bars indicate SDs. Results with statistical significant are marked with asterisks (P< 0.05).

Because PGA was the major component of the biofilm matrix of *A*. *pleuropneumoniae*, we used quantitative reverse-transcriptase PCR (qRT-PCR) to determine whether the expression of *pgaA* and *cpxR* genes was affected in Δ*tolC*. The *pgaA* gene is the first gene in the operon *pgaABCD* that encodes PGA. The *cpxR* gene encodes the response regulator of the CpxAR two component regulatory system, which was previously reported to regulate biofilm formation [16]. When compared against WT, the expression of both *pgaA* and *cpxR* were significantly lower in Δ*tolC* at both 4-h and 6-h (Fig 5A and 5B). However, no significant difference in *pgaA* and *cpxR* transcription levels was observed between the two strains at 12-h (Fig 5C). These results suggested that *tolC* mutation reduced the mRNA levels of *pgaA* and *cpxR* in the early, initial surface adherence stage of biofilm formation, but not at the maturation stage.

### 2.8 Inactivation of TolC results in the secretion deficit of certain biofilm-promoting factor in *A*. *pleuropneumoniae*

**Fig 6:**
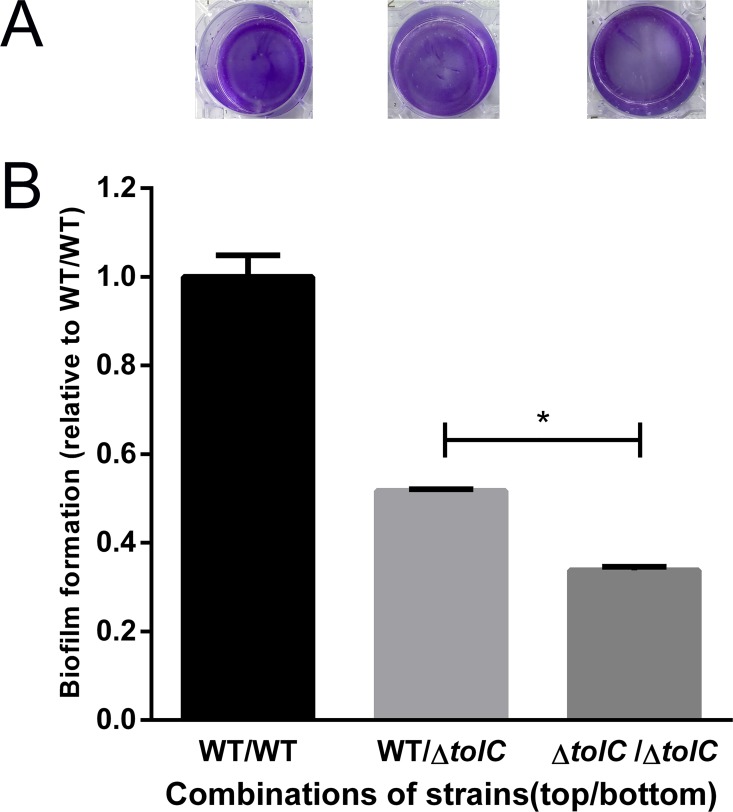
Coculturing with WT rescued biofilm formation defects of Δ*tolC*. Biofilm formation was performed by coculturing Δ*tolC* with WT in Transwell chambers. (A) Biofilms in the lower chamber were stained with crystal violet. (B) Quantitative analysis of biofilms. Data presented here are means of triplicate experiments and error bars indicate the SDs. *, statistical significance (P< 0.05).

To investigate whether the deficiency of Δ*tolC* biofilms can be rescued by the presence of wild-type supernatant, transwell assays were performed. Three combinations of strains—WT/Δ*tolC* mutant, Δ*tolC*/Δ*tolC* and WT/WT (top/bottom)—were used. After 12h of incubation, compared to the Δ*tolC*/Δ*tolC* group, biofilm formation by Δ*tolC* in the WT/Δ*tolC* was significantly rescued, though still below the wild-type level (Fig 6A). The biofilm biomass in the lower chamber was quantified by crystal violet staining. The data were consistent with the observation in the transwell plates (Fig 6B). Together, coculturing with wild-type strain appeared to have the ability to partially restore the ability of Δ*tolC* to form biofilms.

## 3. Discussion

TolC, an outer membrane channel protein, was widely studied for its crucial role in multidrug resistance and virulence in numerous Gram-negative bacteria pathogens [17,20]. TolC was reported to be involved in the biofilm formation of *S*. *typhimurium* and *E*. *coli* [21,22]. Functional TolC proteins have been found in several members of the *Pasteurellaceae* family whose role in antibacterial resistance are well characterized, but the function of TolC in biofilm formation of this group of important pathogens has not been examined.

In this study, we examined the importance of TolC in biofilm formation of *A*. *pleuropneumoniae*. Overall, the time course of biofilm formation in WT in this study is slower than the one previously published by others [16]. The results suggested that the kinetics of biofilm formation varies among different *A*. *pleuropneumoniae* field isolates. Interestingly, while Δ*tolC* showed initial attachment defects, it showed similar biofilm development schedule in which biofilms reached its maximum at 6 h after incubation and remained at 12h. This result suggested that deletion of *tolC* did affect the biofilm maturation but did not affect the biofilm maturation rate in *A*. *pleuropneumoniae*.

Attachment of bacterial cells to abiotic surfaces and aggregation into microcolonies are considered the first step of biofilm formation, and cell surface hydrophobicity and motility play important roles in bacterial attachment [8,23]. When compared against WT, Δ*tolC* had significantly reduced surface attachment by crystal violet staining (Fig 2A), which was confirmed by CLSM images that showed sparse bacteria colonies (Fig 4). We speculated that the loss of an important membrane protein such as TolC from the outer membrane of bacteria probably changed the surface hydrophobicity of the cells. Indeed, the cell hydrophobicity of Δ*tolC* was decreased (Fig 2C). Bacterial motility, a factor that may impact on the adherence of biofilm cells, was also determined using a soft-agar plate as published previously [24]. As a result, the Δ*tolC* displayed the similar motility phenotype with WT (Data not shown).

EPS are known as a conglomeration of different types of biopolymers that account for over 90% of the dry mass of biofilms. The excretion of EPS is critical for the initial adherence as well as biofilm maturation [7]. A previous study suggested that eDNA and proteins contributed little to the biofilm architecture of *A*. *pleuropneumoniae* [25]. Rather, PGA is a major surface polysaccharide that acts as the scaffold of biofilm matrix and functions in intercellular adhesion within the biofilm colony [10]. In this study, PGA was confirmed to be an important fraction of the biofilm matrix of WT strain SC1516. However, we found that eDNA and proteins also contributed to the biofilm architecture of SC1516 (Fig 3). This unexpected result was probably due to that the compositions and ratios of EPS might vary greatly between strains of a single species [7]. When compared against the WT, the biofilm composition of Δ*tolC* was always deficient in the extracellular proteins during the entire process of biofilm formation (Fig 3). One potential mechanism for this phenotype is that inactivation of TolC reduces the export of secreted proteins [26]. Importantly, we showed that Δ*tolC* produced lesser amount of PGA in the initial stage of biofilm formation, as demonstrated by the dispersin B treatment (Fig 3A), and also by the CLSM images (Fig 4). Considering the essential role of PGA in intercellular adhesion within the biofilm colony, we propose that the lower level of PGA production in Δ*tolC* at the initial stage of biofilm formation likely contributes to the reduced surface attachment. Our hypothesis is corroborated by the observation that the expression of *pgaA* was significantly down-regulated in Δ*tolC* at the initial stage of biofilm formation (Fig 5A).

Previous studies have shown that TolC of *E*. *coli* could directly respond to membrane stress [17]. CpxAR is a classical two-component system that is involved in envelope stress response in *E*. *coli* [27]. The link between TolC and CpxAR system has not been reported. Previous transcriptomic analyses of *A*. *pleuropneumoniae* biofilms revealed the important role of *cpxAR* in biofilm growth [16]. Given that regulator sigma factor σ^E^ was one of the Cpx-regulated genes and the *pga* operon was positively regulated by σ^E^ [28], the correlation between TolC, CpxAR system and *pga* operon attracted much interest. We found that *cpxR* was significantly down-regulated in Δ*tolC*. Whether the *pga* operon was positively regulated by the CpxAR, and whether the regulation of biofilm formation by *tolC* is achieved by affecting the expression of *cpxAR*, is currently unknown. Further experiments are needed to clarify the interplays between TolC, CpxAR and PGA biosynthesis.

TolC has been well characterized as the channel of efflux pumps and type I secretion systems, through which a wide range of substrates could be exported, such as antibiotics, toxins and signaling molecules [29,30]. In the transwell assay, TolC of the wild type likely secreted some unknown soluble biofilm-promoting factor(s) that crossfeed and partially restored biofilm formation in Δ*tolC* (Fig 6). This finding was unexpected, because no “rescue” effect was observed during coculturing of a Δ*tolC* mutant of *S*. *typhimurium* with its wild-type parental strain [18]. We speculate that the "biofilm-promoting factor" could be quorum sensing molecules that affect the initial adherence and maturation of biofilms.

On the other hand, we have confirmed that TolC is essential for the secretion of ApxII, an alpha-hemolysin produced by *A*. *pleuropneumoniae* (Data not shown). Previous studies have shown that alpha-toxins are required for biofilm formation of *S*. *aureus* and *Streptococcus pneumoniae* [31,32]. Therefore, it is possible that ApxII toxin is one of the factors secreted through TolC that “rescue” biofilm formation in Δ*tolC*. Further studies are required to clarify the mechanism.

In summary, our current study identified the importance of TolC in the formation of a competent biofilm in *A*. *pleuropneumoniae*. Based on our results, we propose that the absence of TolC impairs biofilm formation by reducing the initial attachment of *A*. *pleuropneumoniae*. Several factors contribute to the reduction of initial biofilm adherence in Δ*tolC*. Among them, the down regulation of *pgaA* expression, consequently, reduced PGA production and the changes in surface hydrophobicity of the Δ*tolC* cells seem to be major contributors. Besides, the reduction of extracellular proteins within biofilm matrix caused by the loss of TolC would also be an additional explanation for the deficiency of biofilm formation. Our findings demonstrate that TolC is essential for the biofilm formation of *A*. *pleuropneumoniae* by impacting upon the initial attachment of biofilm cells. These findings highlight the potential of targeting TolC in the antibiofilm strategies of *A*. *pleuropneumoniae*.

## 4. Materials and Methods

### 4.1 Bacterial strains and growth conditions

*A*. *pleuropneumoniae* wild-type strain SC1516 (serotype 7), an isolate from a diseased pig in Sichuan, China, was used as the parental strain. Δ*tolC* was derived from SC1516, in which the *tolC* gene was deleted. Δ*tolC*/*tolC* was the genetically complementary strain of Δ*tolC*. The Δ*tolC* and Δ*tolC*/*tolC* strains were both constructed in our recent study (Unpublished data). Briefly, an 842-bp DNA fragment obtained from *tolC* and the chloramphenicol acetyl transferase gene were fused by overlap PCR and cloned into a pMD19-T vector. The resulting recombinant plasmid was electroporated into strain SC1516 to construct Δ*tolC* mutant. The full-length *tolC* with its promoter sequence was cloned into the shuttle vector pLS88, followed by electroporation into the Δ*tolC* to generate Δ*tolC*/*tolC*. The WT and its derivatives were cultured in Tryptic Soy Broth (TSB, Difco Laboratories, Detroit, USA) supplemented with 0.01% β-nicotinamide adenine dinucleotide (NAD). Where necessary, 50 μg/ml kanamycin or 2 μg/ml chloramphenicol were added. *E*. *coli* strains were grown in Luria–Bertani (LB, Difco) medium or on LB agar and ampicillin (100 μg/ml) was added when necessary. All strains were grown at 37°C.

### 4.2 Biofilm formation and surface attachment assays

To analyze the time course of biofilm formation, overnight broth cultures of WT, Δ*tolC* and Δ*tolC*/*tolC* were inoculated into fresh TSB medium by dilution of 1:100 and transferred to the 96-well polystyrene microtiter plates in triplicate (Costar 3599, Corning, NY, USA) for 0, 4, 6, 12, 24 and 36 h at 37°C, respectively. Biofilms were washed with water and stained with 100 μl of 0.1% crystal violet for 5 min at room temperature. After washing and drying, 100 μl of acetic acid (33%, v/v) was added to each well to release the bound dye. The optical density at 595 nm was measured using a microplate reader (Bio-Rad iMark^TM^ microplate Reader).

For the surface attachment assays, bacteria from overnight cultures were inoculated with a dilution (1/100) into a 96-well microtiter plate in triplicate for 4h at 37°C. Attached cells in the wells were stained with crystal violet and quantified as described above. The OD 595 nm is considered to be proportional to the number of attached cells in the well [10].

### 4.3 Autoaggregation assay

The abilities of *A*. *pleuropneumoniae *strains to autoaggregate were evaluated as previously described with minor modifications [33]. Briefly, single colonies of the WT, Δ*tolC* and Δ*tolC*/*tolC* were cultured in 3 mL of TSB medium with agitation for 12h. Then, cultures were allowed to remain static at room temperature for 6h and photographed.

### 4.4 Cell surface hydrophobicity assay

Hydrophobicity of a bacterial surface was determined as described previously [34], with minor modifications. In brief, cells of WT, Δ*tolC* and Δ*tolC*/*tolC* were harvested from TSB cultures (OD550 = 0.8) by centrifugation at 5000 rpm for 3 min, respectively. Pellets were washed twice with PUM buffer and resuspended in the same buffer. 1 ml of suspensions was mixed with 200 μl of hexadecane, by vortex for 1 min. The phases were allowed to separate for 30 min at 30°C. The hydrophobicity indexes were determined by the equation: [(A_0_-A) A_0_^-1^] ×100, as described previously [23]. A_0_ and A were the initial and final optical densities of the aqueous phase at A550 nm, respectively. This experiment was repeated three times independently.

### 4.5 Expression and purification of *A*. *pleuropneumoniae* dispersin B

Genomic DNA of *A*. *pleuropneumoniae* SC1516 was amplified by PCR using the forward primer 5’- CATGCCATGGCAACTTATGCAAACGCTATGG-3’ and the reverse primer 5’-CCGCTCGAGATGCG ATTTCGGATCATTAG-3’. The restriction sites were underlined in each primer sequence. The PCR product was digested with NcoI and XhoI followed by ligation into the NcoI/XhoI sites of the expression vector pET-22b (+). The resulting recombinant plasmid pEDB was transformed into *E*. *coli* BL21 (DE3). Expression of dispersin B was induced by the addition of 0.5 mM isopropyl-beta-D-thiogalactopyranoside (IPTG) and the recombinant protein was purified from the supernatant by Ni affinity chromatography using Profinity IMAC Ni-Charged Resin (Biorad) as described previously [35]. The concentration of dispersin B was indirectly quantified using a Bradford assay as previously described [36].

### 4.6 Enzymatic treatments

Biofilm dispersion assays were performed on WT and Δ*tolC* as previously described [37]. Biofilms were cultured in a 96-well microtiter plate as described above for 4h, 6h and 12h at 37°C. Then, 100 μg/ml of dispersin B, 500 μg/ml of DNase I and 500 μg/ml of proteinase K were added to the biofilms, respectively, 50 μl per well. Meanwhile, control wells were added with 50 μl of Tris-HCl buffer without the enzyme. Wells with dispersin B were treated for 5 min, and those with DNase I and proteinase K were treated for 1 h at 37°C. After the incubation, biofilms were washed, stained with crystal violet, and quantified as described above. This experiment was repeated for three times independently with triplicate samples.

### 4.7 Confocal laser scanning microscopy

Static biofilms were cultured on 20-mm^2^ coverglasses submerged in 3 ml TSB broth at the bottom of 6-well microtiter plate for 4 h or 6 h at 37°C, respectively. Then, the supernatants were removed and the biofilms were washed with water and stained with LIVE/DEAD^@^ BacLight^Tm^ Bacterial Viability Kit (catalog no. L13152; Molecular Probes, Invitrogen, USA) or Wheat Germ Agglutinin (WGA)–Oregon Green^@^ 488 (Invitrogen, Molecular Probes, OR, USA) according to the manufacturer’s instructions. Stained biofilms were visualized with a Nikon A1R confocal scanning laser microscope and images were acquired using the NIS-Elements AR software (Nikon, Japan).

### 4.8 RNA extraction and qRT-PCR

For qRT-PCR, WT and Δ*tolC* were grown in TSB in 6-well plates for 4h, 6h or 12h, respectively. After the removal of supernatants, biofilms were collected with a cell scraper. Cell pellets were resuspended in PBS with 50% ice-cold methanol to prevent changes in transcript levels. RNA extraction and reverse transcription of total RNA were performed as described before [38]. 50-fold dilutions of cDNA were used as the template for qRT-PCR reactions in triplicate using the MiniOpticon™ Real-Time PCR Detection System (Bio-Rad, USA). Relative expression was normalized against the 16S rRNA gene, and the data were calculated with a threshold cycle (ΔΔCt) method as previously described [13]. Primers used were as follows: *pgaA*, AAGCGGTTGCCGTGTTAG and ACGTTTGCTCGGTCATGG; *cpxR*, GGGCAAATTCTTTCTCGTG and AAACCAAGGCA AGTTATCGT; *16s*, ACCCTTATCCTTTGTTGC and CATCTTGCTTCCCTCTGT. This experiment was repeated for three times independently with triplicate samples.

### 4.9 Transwell assays

A transwell assay was carried out using the 6-well polystyrene transwell plate with a 0.4-μm-pore-size polyester membrane (Corning, NY). With this permeable membrane, secretions or metabolite, but not bacterial cells in the supernatant of the upper chamber were allowed into the lower chamber where the biofilm formed. 3 ml of TSB broth was inoculated with 30 μl of an overnight culture of Δ*tolC* in the lower chamber, and 1 ml WT suspension (1/100 dilution) in the upper chamber. Control wells were set with WT or Δ*tolC* suspension in both top and bottom wells. Transwell plates were incubated at 37°C for 12h. Bacterial suspension in the upper chamber was removed and the biofilms formed in the lower chamber were stained and quantified using crystal violet staining. This experiment was repeated independently three times with triplicate samples.

### 4.10 Statistical analysis

Data were analyzed with GraphPad Prism version 6.0 (GraphPad Software, San Diego, CA). One-way analysis of variance (ANOVA) test was used for surface attachment and transwell assays. Two-way ANOVA test was used for biofilm formation, enzymatic treatments and qRT-PCR analysis. P values of < 0.05 were considered significant and indicated by an asterisk.
